# Acute Resistance Exercise Temporarily Reduces Circulating Adiponectin in Trained Young Men: A Pilot Study

**DOI:** 10.3390/biom16020229

**Published:** 2026-02-02

**Authors:** Luigi Marano, Marta Mallardo, Ersilia Nigro, Furqan Memon, Viktoriia Fylymonenko, Eleonora Martegani, Sara Missaglia, Ferdinando Cereda, Daniela Tavian, Aurora Daniele

**Affiliations:** 1 Laboratory of Cellular Biochemistry and Molecular Biology, CRIBENS (Center of Research in Biochemistry and Nutrition of Sport), Catholic University of Sacred Heart, 20145 Milan, Italy; luigi.marano@unicatt.it (L.M.); viktoriia.fylymonenko@unicatt.it (V.F.); eleonora.martegani@unicatt.it (E.M.); sara.missaglia@unicatt.it (S.M.); 2 CEINGE (Centro Ingegneria Genetica) Biotecnologie Avanzate “Franco Salvatore” Scarl, Via G. Salvatore 486, 80145 Napoli, Italy; marta.mallardo@unipegaso.it (M.M.); ersilia.nigro@unicampania.it (E.N.); furqan.memon@unicampania.it (F.M.); aurora.daniele@unina.it (A.D.); 3 Department of Education and Sport Sciences, Pegaso Telematic University, 80143 Naples, Italy; 4 Dipartimento di Scienze e Tecnologie Ambientali, Biologiche, Farmaceutiche, Università della Campania “Luigi Vanvitelli”, Via A. Vivaldi, 81100 Caserta, Italy; 5 Department of Psychology, Catholic University of Sacred Heart, 20123 Milan, Italy; 6 Department of Education, Catholic University of Sacred Heart, 20123 Milan, Italy; ferdinando.cereda@unicatt.it; 7 Dipartimento di Medicina Molecolare e Biotecnologie Mediche, Università degli Studi di Napoli “Federico II”, Via Pansini, 80131 Napoli, Italy

**Keywords:** adiponectin, resistance exercise, time under tension, creatine kinase, exercise metabolism

## Abstract

Background: Adiponectin is an adipokine with insulin-sensitizing, anti-inflammatory, and cytoprotective properties that also plays a key role in metabolic adaptation to exercise. Although its regulation after resistance exercise has been extensively documented, less is known about its short-term modulation and its correlation with muscle damage markers following resistance training. Methods: Nine resistance-trained young men completed two sessions of total-body resistance exercise: (1) high time under tension (TUT) (5-1-2-1 cadence, to failure; ETS1) and (2) moderate TUT (2-1-2-1 cadence, two repetitions in reserve; ETS2). Plasma and saliva samples were collected before exercise and at 15 min, 24 h, and 48 h after exercise to assess total adiponectin by ELISA. Plasma creatine kinase (CK) and a Visual Analog Scale (VAS) were also measured for muscle soreness. Results: Plasma adiponectin significantly decreased from baseline to 48 h post-exercise in both sessions (*p* < 0.001), with no differences between the TUT conditions. Salivary adiponectin remained unchanged. Although a significant increase in CK and a decrease in adiponectin were observed at the group level, correlation analysis revealed no significant linear relationship between the magnitude of CK elevation and adiponectin reduction. Conclusions: Overall, these findings support the role of adiponectin as a marker of acute metabolic adaptation to resistance exercise. Acute resistance exercise elicited a time-dependent decrease in circulating adiponectin, irrespective of TUT. The temporal pattern of adiponectin decrease coincided with the rise in muscle damage markers, yet the lack of direct correlation suggests distinct regulatory mechanisms, while the lack of salivary changes underscores the complexity of adipokine regulation in vivo and suggests that saliva is not a reliable indicator of changes in circulating adiponectin.

## 1. Introduction

Adiponectin is a pleiotropic adipokine predominantly secreted by adipose tissue that plays a central role in the regulation of systemic energy homeostasis, insulin sensitivity, and inflammatory balance [1,2]. Beyond its classical metabolic actions, adiponectin acts as a key molecular mediator of exercise-induced adaptations by modulating intracellular signaling pathways involved in fatty acid oxidation, glucose uptake, mitochondrial biogenesis, and cellular stress resistance [3,4]. These effects are mediated primarily through the activation of AdipoR1 and AdipoR2 adiponectin receptors, which are highly expressed in skeletal muscle and liver. Through these mechanisms, adiponectin contributes to the coordination of substrate utilization during periods of increased energetic demand [5].

Regular exercise is essential to maintain health and prevent disease, improve cardiovascular function, insulin sensitivity, and body composition [6,7,8]. It represents a potent physiological stimulus capable of acutely and chronically modulating adiponectin signaling. During exercise, adiponectin has been shown to facilitate exercise-induced improvements in insulin sensitivity and oxidative metabolism, while also exerting cytoprotective and anti-inflammatory effects that support tissue adaptation and repair [9]. However, emerging evidence suggests that adiponectin responses to exercise are highly context-dependent, influenced by factors such as exercise intensity, duration, metabolic load, and recovery dynamics. Understanding the regulation of the endocrine functions of adipose tissue during and after exercise is crucial for developing targeted interventions to promote health and longevity.

Data from the literature reported that chronic resistance exercise generally increases basal adiponectin concentrations, while acute responses to exercise are less consistent and appear to be influenced by the modality, intensity, and duration of exercise [10,11]. Recent evidence from animal studies has highlighted the active role of adiponectin in recovery and cellular protection [12]. In particular, Wu et al. (2024) demonstrated that exercise-induced adiponectin secretion enhances autophagic flux and protects hepatocytes from lipid toxicity. This link between adiponectin signaling, autophagy, and energy metabolism underscores its role as a crucial mediator of adaptation to exercise [13].

Resistance exercise triggers both mechanical and metabolic stress, stimulating muscle hypertrophy, remodeling, and endocrine responses. Among resistance training variables, time under tension (TUT) modulates contraction velocity and metabolic load, thus influencing acute molecular responses [14,15]. However, the acute impact of different TUT programs on the dynamics of circulating adiponectin remains unexplored. Understanding how different TUT protocols influence adipokine responses could help to clarify the relationship between exercise intensity, metabolic signaling, and recovery mechanisms. Metabolic stress represents a central determinant of endocrine responses to exercise, independently of the specific training modality. High-intensity interval training (HIIT) is well recognized for inducing pronounced metabolic perturbations associated with reductions in circulating adiponectin during the early recovery phase [16]. Notably, similar metabolic conditions may also arise during resistance exercise when TUT is prolonged, particularly through extended eccentric contractions and reduced intramuscular perfusion. Both HIIT and prolonged TUT resistance protocols may converge on similar regulatory pathways, potentially leading to comparable adiponectin regulation.

These similar regulatory patterns have been described after endurance exercise protocols with high time under load, suggesting that adiponectin may act during acute overload to prioritize substrate availability and restore it once homeostasis is re-established [17].

Today, the determinants of the regulation of adiponectin remain incompletely understood. The available literature rarely integrates responses of adipose tissue with markers of muscle damage and recovery, limiting the interpretation of adiponectin fluctuations within the broader stress–recovery framework. The absence of studies directly comparing resistance exercise protocols differing in TUT but matched for load and volume represents a critical gap in understanding whether adiponectin responses are driven by mechanical factors or by the magnitude of metabolic stress per se.

In this context, our study aimed to characterize the time-dependent changes in plasma and salivary adiponectin after two resistance training sessions differing in TUT. We hypothesized that both protocols would modulate plasma concentrations of adiponectin. Secondary analysis examined the relationships among adiponectin and CK as biomarkers of metabolic and muscular stress.

## 2. Materials and Methods

### 2.1. Participants’ Recruitment

Nine healthy male participants were recruited for this study. Participants were recreationally resistance-trained men and were not competitive/elite athletes at the time of enrollment. To be included, participants had to be considered resistance-trained, defined as (a) having a minimum of two years of continuous resistance training experience; (b) training consistently at a frequency of at least three times per week; and (c) demonstrating a baseline level of strength, such as a 1RM in the leg press equal to at least 1.5 times their body weight. Exclusion criteria were any musculoskeletal injury in the last 6 months, the use of anabolic drugs or supplements, or medical conditions that could interfere with the study outcomes. It should be noted that while the mean BMI of the cohort approached the conventional overweight threshold (24.5 kg/m^2^), the participants were not selected based on BMI, and body composition indicated low adiposity and high muscularity (fat mass 14.8% and skeletal muscle mass 44.5%; Table 1), suggesting that BMI in this cohort primarily reflected fat-free mass.

Anthropometric measurements were obtained: height was recorded to the nearest 0.1 cm using a calibrated stadiometer (Seca 217, Seca GmbH & Co. KG, Hamburg, Germany), and body weight was measured to the nearest 0.1 kg using a mechanical scale (Seca Viva 750, Seca GmbH & Co. KG, Hamburg, Germany). Body composition and hydration status were evaluated using bioelectrical impedance analysis (BIA) via the Akern BIA 101 BIVA^®^ PRO IPS (ItaAkern s.r.l., Florence, Italy). All measurements followed the manufacturer’s instructions [18,19]. The anthropometric characteristics of the study’s participants are presented in Table 1. Blood samples were collected using a Vacutainer system. Immediately after collection, the samples were centrifuged at 11,000 rpm for 10 min. Plasma was carefully separated from cellular components and stored at −80 °C until further analysis. For saliva collection, participants were instructed to rinse their mouths carefully five times without swallowing to remove food residues and contaminants. Subsequently, they deposited saliva into 50 mL sterile Falcon tubes. These samples were centrifuged at 2500 rpm for 4 min, and the supernatant was stored at −80 °C until analysis.

All experimental procedures in this study adhered to the principles outlined in the Declaration of Helsinki and received approval from the Institutional Ethics Committee of the Department of Psychology at the Catholic University of the Sacred Heart of Milan (CERPS), under protocol number 36/24. The approval date for the specific project is the 5 February 2024. All participants provided their written informed consent prior to enrollment.

### 2.2. Experimental Procedures

**Figure 1:**
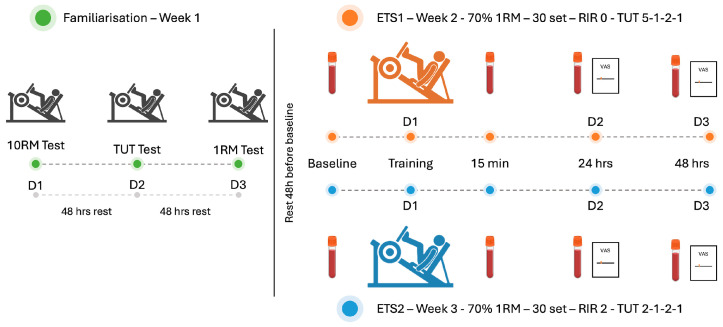
Experimental procedures flow chart.

Figure 1 illustrates the experimental timeline and procedures used. The details of the exercise protocol are available on request.

All assessments and training were carried out in a private gym facility where environmental parameters were regulated, maintaining a temperature between 18 °C and 22 °C and relative humidity below 60%. To minimize the influence of coordination and skill acquisition, all exercises were performed using isotonic resistance machines. To control confounding variables, individuals were instructed to maintain regular sleep routines and avoid the intake of alcohol, caffeine, or stimulants in the hours preceding each visit. All training and data collection sessions were scheduled between 09:00 and 13:00 to minimize circadian influences on physiological responses. Participants were instructed to stay at rest for at least 48 h before baseline sample collection and to avoid any form of exercise for 2 days after performing the testing sessions.

Biological samples, blood and saliva, were obtained at four specific intervals relative to the exercise sessions: before exercise (baseline), and then at 15 min, 24 h, and 48 h post-exercise. Plasma and salivary adiponectin concentrations, as well as plasma creatine kinase (CK), were analyzed. In addition, participants completed the Visual Analogue Scale (VAS) at the 24 and 48 h post-exercise time points to subjectively rate muscle soreness. 

### 2.3. Details of Experimental Testing Sessions

Table 2 details the specific exercises performed during both Experimental Testing Session 1 (ETS1—high TUT, 5-1-2-1 cadence, to failure) and Experimental Testing Session 2 (ETS2—moderate TUT, 2-1-2-1 cadence, submaximal effort), highlighting the execution modalities specific to each session.

For both sessions, the load was set at 70% of each participant’s individual 1RM, as determined during familiarization based on established guidelines [19]. Prior to each experimental testing session (ETS1 and ETS2), participants completed a standardized warm-up. In addition, the time under tension protocol used in ETS1 was designed to elicit greater metabolic stress, primarily through the prolonged eccentric phase, by inducing transient local muscle ischemia and promoting metabolite accumulation [20]. On average, participants completed approximately 223 (±30.5) repetitions during ETS1 and 248 (±44.3) during ETS2. The calculated training load, expressed in arbitrary units (AU) as the product of load-kg-repetitions, and sets, averaged 123,231.44 ± 28,057.40 AU for ETS1 and 137,708.29 ± 36,408.27 AU for ETS2. Each session was closely supervised by a qualified trainer who ensured strict adherence to the prescribed repetition tempo, rest intervals, and execution technique.

### 2.4. Adiponectin and CK Dosage

The concentration of plasma and salivary total adiponectin was determined using an enzyme-linked immunosorbent assay (ELISA) with a polyclonal antibody developed in-house against a human adiponectin amino acid fragment (H2N-ETTTQGPGVLLPLPKG-COOH), as previously described [21]. Each sample was analyzed in duplicate, and two independent measurements were performed for accuracy. CK levels were measured using a Chemiluminescent Immunoassay method (CLIA, Siemens Healthcare Diagnostics, Tarrytown, NY, USA).

### 2.5. Adiponectin Oligomers Evaluation by Western Blotting

Five microliters of plasma were treated with 1 × Laemmli buffer, heated to 95 °C for 2 min, loaded under non-reducing conditions on 10% SDS-PAGE gel, and transferred as previously described [21]. The blots were scanned using the ChemiDoc MP imaging system (Bio-Rad, Hercules, CA, USA) and analyzed by carrying out densitometry with ImageJ software V 1.53.

### 2.6. Statistics

Statistical analyses were conducted using JASP software (Version 0.19.1; JASP Team, 2024, The Netherlands). Statistical significance was set at *p* < 0.05 (two-tailed). Data normality was assessed using the Shapiro–Wilk test, together with evaluations of skewness and kurtosis indices and visual inspection of distribution histograms. Because the assumption of normality was violated for several dependent variables (*p* < 0.05), non-parametric tests were used for inferential analyses.

The sample size was estimated a priori using G*Power (Version 3.1.9.6) based on a repeated-measures ANOVA framework (within-between interaction) as a planning benchmark. Assuming a medium effect size (f = 0.40), α = 0.05, and power (1 − β) = 0.80, the required sample size was 12 participants; due to recruitment constraints, nine participants completed the study. To provide quantitative context for interpretation given *n* = 9, we performed sensitivity analyses in G*Power (Version 3.1.9.6) to estimate the minimum detectable effects under the stated assumptions, following recent methodological recommendations [22]. For the effect of within-session omnibus time across four repeated measures (one group; four measurements), the inputs were α = 0.05 (two-tailed), power (1 − β) = 0.80, *n* = 9, assumed average correlation between repeated measures r = 0.50, and non-sphericity correction ε = 1.0, resulting in a minimum detectable effect of f = 0.42. For between-protocol contrasts, the sensitivity analysis targeted the four paired comparisons between ETS1 and ETS2 at matched time points (baseline, 15 min, 24 h, 48 h) using a matched-pairs model (two-tailed). Using a conservative familywise α = 0.0125 (0.05/4) with a power (1 − β) = 0.80, and *n* = 9, a minimum detectable paired effect of dₙ (dz) = 1.39 yielded (for reference, a single paired comparison α = 0.05 yielded dz = 1.07). These sensitivity analyses provide quantitative context for interpreting non-significant ETS1 vs. ETS2 contrasts and do not replace the non-parametric inferential analyses described below.

Changes in adiponectin and creatine kinase (CK) levels over time within ETS1 and within ETS2 were analyzed using the Friedman test. When a significant omnibus effect was detected, Conover post hoc comparisons were used to identify specific time-point differences. Pairwise comparisons between ETS1 and ETS2 at matched time points were analyzed using the Wilcoxon signed-rank test. Visual Analogue Scale (VAS) scores were analyzed using the Wilcoxon signed-rank test to assess changes between measurement points. The correlations were assessed using Spearman’s rank correlation coefficient (ρ). The correlation strength was interpreted as negligible (0.00–0.10), weak (0.10–0.39), moderate (0.40–0.69), strong (0.70–0.89), and very strong (0.90–1.00).

Effect sizes are reported as Kendall’s *W* for Friedman tests and matched rank-biserial correlation (*r_r_b*) for Wilcoxon tests, with confidence intervals (95%) where applicable.

## 3. Results

### 3.1. Plasma Adiponectin Decreases in a Time-Dependent Manner Following Both Training Protocols, While Salivary Levels Do Not Change

**Figure 2:**
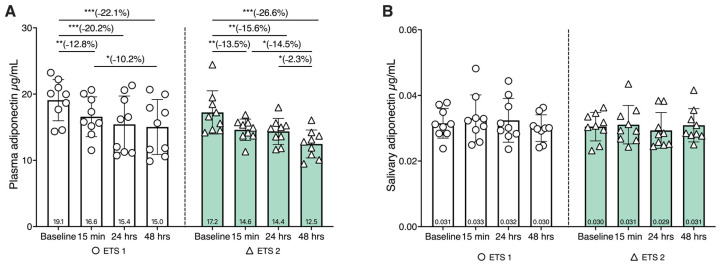
Plasma and salivary adiponectin concentrations at baseline and 15 min, 24 h, and 48 h after the two exercise training sessions (ETS1 and ETS2). (**A**) Plasma levels of adiponectin after ETS1 (white bars) and ETS2 (green bars). Within-session differences across time were assessed using the Friedman test (ETS1: *χ*^2^*F*(3) = 16.6, *p* < 0.001, Kendall’s *W* = 0.615; ETS2: *χ*^2^*F* (3) = 13.4, *p* = 0.004, Kendall’s *W* = 0.496). Conover post hoc comparisons (two-tailed) versus baseline showed lower plasma adiponectin at 15 min (ETS1: *p* = 0.003, *r_r_b* = 0.911; ETS2: *p* = 0.013, *r_r_b* = 0.911), 24 h (ETS1: *p* < 0.001, *r_r_b* = 1.000; ETS2: *p* = 0.013, *r_r_b* = 0.956), and 48 h (ETS1: *p* < 0.001, *r_r_b* = 1.000; ETS2: *p* < 0.001, *r_r_b* = 0.956). (**B**) Salivary adiponectin levels after ETS1 and ETS2; no time effect was detected (ETS1: *χ*^2^*F*(3) = 3.533, *p* = 0.316, Kendall’s *W* = 0.131; ETS2: *χ*^2^*F*(3) = 2.733, *p* = 0.435, Kendall’s *W* = 0.101). Data are expressed as means ± SD. * *p* < 0.05; ** *p* < 0.01; *** *p* < 0.001. **Note.** *χ*^2^*F* denotes the Friedman chi-square statistic (df = 3), and Kendall’s *W* is reported as the effect size for the Friedman test, representing the degree of concordance among repeated measures (0–1). Conover post hoc comparisons were performed following the Friedman test (grouped by subject). The effect size for post hoc contrasts is reported as the matched rank-biserial correlation (*r_r_b*), derived from paired signed-rank tests. *p*-values are two-tailed.

To investigate the possible modulation of adiponectin expression in response to both ETS1 and ETS2, plasma and salivary adiponectin were evaluated at baseline, at 15 min, at 24 h, and at 48 h post-exercise (Figure 2, panel A). In both training protocols, plasma adiponectin levels showed a significant, time-dependent decrease from baseline to 48 h after exercise. In particular, plasma adiponectin decreased by approximately 15–20% at 24 h and by nearly 25% at 48 h compared to baseline values, with the most pronounced reduction observed at the final time point (*p* < 0.05); this trend was similar across the two exercise protocols. In contrast, salivary adiponectin remained unchanged throughout the different time points (Figure 2, panel B).

### 3.2. HMW Adiponectin Oligomers Decrease Following Both Training Protocols

**Figure 3:**
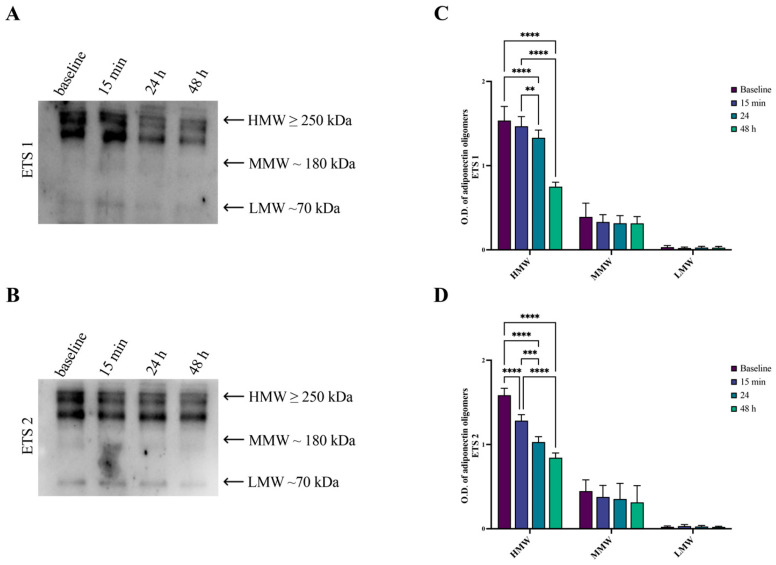
Western blot analysis of adiponectin oligomers in plasma from subjects involved in the study at baseline and 15 min, 24 h, and 48 h after the training protocols. Representative Western blot images show the oligomeric distribution of adiponectin [HMW (≥250 kDa), MMW (180 kDa), and LMW (70 kDa)] of a subject at baseline, 15 min, 24, and 48 h after ETS 1 (**A**) and ETS 2 (**B**). The graphic representation of the pixel quantization of all analyzed subjects at baseline and post-exercise time points for ETS1 (**C**) and ETS2 (**D**) was performed by densitometric analysis with the ImageLab software V 1.53. HMW: ** *p* < 0.01; *** *p* < 0.001; **** *p* < 0.001.

Since adiponectin circulates as oligomers of different molecular weights, with HMW oligomers being the most biologically active form, we analyzed the oligomeric distribution to investigate whether the observed changes were primarily related to HMW modulation. Plasma analysis shows different bands corresponding to HMW (≥250 kDa), MMW (≥180 kDa), and LMW (≥70 kDa) oligomers (Figure 3, panel A; Figure S1A). Our data confirmed that plasma adiponectin levels are statistically decreased over time with a specific regard to HMW oligomers, as indicated by the densitometric analysis (Figure 3, panel B; Figure S1B), suggesting that exercise-induced adiponectin changes involve the most active adiponectin isoforms.

### 3.3. Plasma Creatine Kinase Increases Transiently Following Both Training Protocols

**Figure 4:**
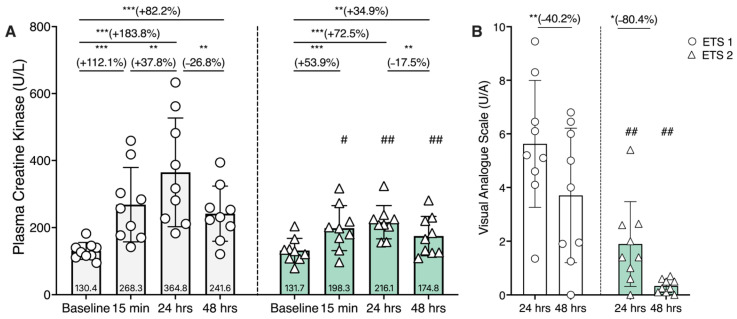
Plasma creatine kinase (CK) concentrations and muscle soreness after the two exercise training sessions (ETS1 and ETS2). (**A**) Plasma CK at baseline, 15 min, 24 h, and 48 h after ETS1 (white bars) and ETS2 (green bars). Within-session differences across time were assessed using the Friedman test (ETS1: *χ*^2^*F*(3) = 19.933, *p* < 0.001, Kendall’s *W* = 0.738; ETS2: *χ*^2^*F*(3) = 17.267, *p* < 0.001, Kendall’s *W* = 0.640). Conover post hoc comparisons (two-tailed, uncorrected) indicated higher CK versus baseline at 15 min, 24 h, and 48 h for both protocols (all *p* ≤ 0.002; *r_r_b* ≥ 0.956). Between-session differences (ETS1 vs ETS2) at each time point were assessed using paired Wilcoxon signed-rank tests; EST2 differed from ETS1 at 15 min (# *p* = 0.027), 24 h (## *p* = 0.008) and 48 h (## *p* = 0.004). (**B**) Muscle soreness was assessed by the Visual Analog Scale (VAS) at 24 h and 48 h after ETS1 and ETS2. Within each protocol, VAS decreased from 24 h to 48 h (ETS1: Wilcoxon *W* = 42, *p* = 0.020, *r_r_b* = 0.867, 95% CI 0.526–0.968; ETS2: *W* = 44, *p* = 0.013, *r_r_b* = 0.956, 95% CI 0.820–0.990). Between-session differences (ETS1 vs ETS2) at each time point were assessed using paired Wilcoxon signed-rank tests; EST2 differed from ETS1 at 24 h and 48 h (## *p* = 0.008). Data are expressed as means ± SD. * *p* < 0.05; ** *p* < 0.01; *** *p* < 0.001. **Note.** *χ*^2^*F* denotes the Friedman chi-square statistic (df = 3), and Kendall’s *W* is reported as the effect size for the Friedman test (0–1). Conover post hoc comparisons were performed following the Friedman test (grouped by subject). Effect size for post hoc contrasts is reported as the matched rank-biserial correlation (*r_r_b*), derived from paired signed-rank tests. *p*-values are two-tailed.

Creatine kinase (CK) activity was measured at baseline, at 15 min, at 24 h, and at 48 h after exercise to assess muscle stress induced by the two training protocols (Figure 4). In both ETS1 and ETS2, CK levels showed a marked increase at 24 h post-exercise compared to baseline (*p* < 0.05). This increase was transient, as CK concentrations declined toward baseline values by 48 h (Figure 4, panel A). In panel B, the VAS indicates a reduction in muscle soreness between 24 and 48 h post-exercise. As expected, VAS and CK were higher after ETS1; VAS values followed the trend of CK (increasing until 24 h and then decreasing), suggesting that the subjects were in a recovery phase. In general, the observed kinetics indicate an acute, exercise-induced muscular response consistent with transient muscle membrane damage.

### 3.4. Adiponectin Levels Correlate with BMI, Body Composition, and Distribution of Body Fluid

**Figure 5:**
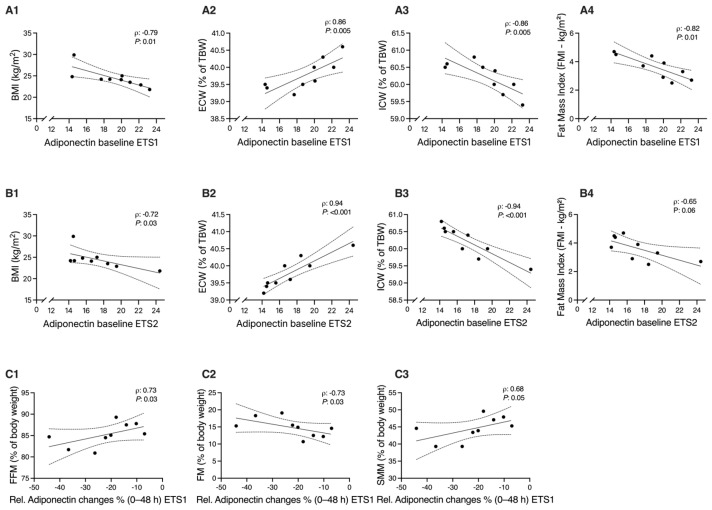
**Correlations between adiponectin and body composition variables.** Panel (**A**) shows correlations between baseline adiponectin concentration and body composition variables for ETS1 ((**A1**) BMI, (**A2**) ECW, (**A3**) ECW, and (**A4**) FMI); panel (**B**) shows corresponding correlations for ETS2 ((**B1**) BMI, (**B2**) ECW, (**B3**) ICW, and (**B4**) FMI). Panel (**C**) illustrates correlations between relative adiponectin changes (%Δ, 0–48 h post-exercise following ETS1) and body composition parameters ((**C1**) FFM%, (**C2**) FM%, and (**C3**) SMM%). ρ (rho) and *p*-values represent Spearman’s rank correlation coefficients. BMI, body mass index; FMI, fat mass index; FM, fat mass; FFM, fat-free mass; SMM, skeletal muscle mass; ECW, extracellular water; ICW, intracellular water. The y-axis is truncated (with an axis break). No observations fall below the lower bound, minimum, ETS1: 14,360 µg/mL; ETS2: 14,190 µg/mL.

**Figure 6:**
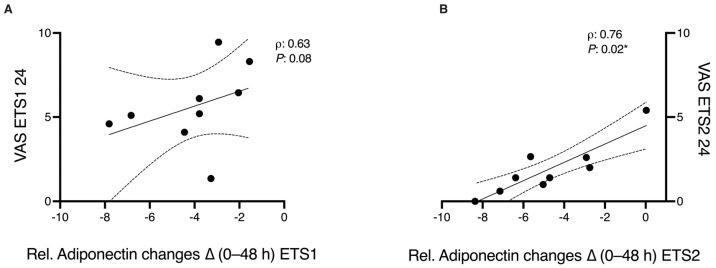
Correlations between Visual Analogue Scale (VAS) 24 h post-exercise and the relative changes in plasma adiponectin (Δ baseline–48 h) following ETS1 (Panel (**A**)) and ETS2 (Panel (**B**)). Spearman’s rank correlation coefficients (ρ) and *p*-values are reported (* *p* < 0.05).

Adiponectin levels at baseline negatively correlate with BMI values for both ETS1 and ETS2 (Figure 5, panels A1 and B1). ECW was directly correlated with adiponectin levels at baseline for both ETS1 and ETS2 (Figure 5, panels A1 and B1), while ICW was inversely related (Figure 6, panels A3 and B3). Similarly, FM was inversely associated with baseline adiponectin but only within ETS1. When we considered delta adiponectin (Adiponectin% bas/48 h), we found correlations with body composition in the ETS1 subgroup. In detail, we found a positive correlation with FFM and SMM while a negative one with FM, suggesting that adiponectin reduction following ETS1 is closely related to the % of lean mass (Figure 5, panels C1, C2, C3). Spearman’s rank correlation analysis revealed no significant associations between absolute or relative changes in plasma adiponectin and CK concentrations at any time point (all *p* > 0.05), indicating that the magnitude of muscle damage did not linearly predict the extent of adiponectin reduction. As shown in Figure 6, VAS scores at 24 h post-exercise showed a positive correlation with adiponectin changes following both training sessions, with a trend toward significance for ETS1 and a significant association for ETS2.

## 4. Discussion

The present study investigated changes in plasma and salivary adiponectin following resistance exercise under two distinct TUT conditions in trained healthy men. The primary finding was a significant time-dependent decrease in plasma adiponectin within 48 h after, regardless of the duration of the TUT, while salivary adiponectin remained unchanged. These results indicated that the regulation of adiponectin in response to acute resistance training is systemic and transient, reflecting metabolic stress and subsequent recovery processes rather than protocol-specific effects. Interestingly, while we observed significant post-exercise CK elevations indicative of heightened metabolic and mechanical stress, these increases were not linearly correlated with the magnitude of plasma adiponectin reductions at the individual level. This reduction in circulating levels is consistent with previous evidence showing a reduction in adiponectin under conditions of acute metabolic overload [23]. Additionally, baseline adiponectin levels showed significant correlations with anthropometric and body composition parameters under both training conditions. In contrast, when considering the change in adiponectin over time (adiponectin% baseline/48 h), significant correlations were found only within the ETS1 subgroup: reductions in adiponectin were positively related to FFM and SMM, and negatively related to FM. These findings suggested that the subjects with a higher proportion of lean tissue showed a greater post-exercise reduction in adiponectin levels. The lack of significant correlations in the ETS2 subgroup may be attributable to the lower training load of this protocol, rather than differences in the recovery period, which was the same between conditions. Accordingly, Barroso et al. reported that changes in plasma adiponectin are strictly related to the reduction in body fat and the increase in lean mass in trained men [24]. Regarding the VAS scores, we found a significant positive correlation with adiponectin levels in the ETS2 subgroup. A trend towards significance was also observed for the ETS1.

The decline in adiponectin levels after exercise may represent a temporary redistribution or consumption of adiponectin within a number of tissues undergoing increased metabolic demand, such as skeletal muscle and liver. Such a decrease in adiponectin is consistent with previous studies that describe transient reductions after high-intensity or exhaustive exercise [25,26]. Accordingly, Wu et al. (2024) demonstrated that exercise promotes activation of adiponectin in hepatocytes [13]. Our results suggested that while acute resistance exercise triggers both CK release and adiponectin clearance, the latter may act as a systemic threshold response rather than being dose-dependent on the magnitude of muscle damage. It is plausible that, in skeletal muscle, acute metabolic stress locally stimulates adiponectin signaling, reducing its circulating pool.

Despite the two TUT programs differing in contraction rates and levels of fatigue, adiponectin exhibited a uniform time-dependent pattern, suggesting that the amount of metabolic stress, rather than the contraction rate itself, drives the hormonal response. This supports previous evidence that adiponectin responses depend more on total energy expenditure and intensity than on exercise modality [10,11]. It is likely that both the high and moderate TUT training protocols produced similar metabolic changes due to the corresponding total training loads.

New evidence is expanding the role of adiponectin beyond its classic metabolic actions, including its integration into cross-talk between muscle and adipose tissue and creatine turnover [27]. Recent studies demonstrated that adipose tissue expresses the transporter of creatine and participates in the regulation of thermogenesis and whole-body energy expenditure [27]. In this context, adiponectin can be considered not only an indicator of metabolic and inflammatory health but also a regulator of post-exercise energy redistribution. Our hypothesis is that increased metabolic stress and inflammatory responses during muscle repair may contribute to the transient downregulation of circulating adiponectin. Recently, plasma adiponectin downregulation has been shown to accompany early recovery phases characterized by oxidative stress and energetic imbalance [27,28].

Although saliva has been proposed as a potential non-invasive biomarker, it may not be a reliable indicator of changes in circulating adiponectin after resistance exercise due to the lack of changes in salivary adiponectin expression observed in the present study [29]. This discrepancy may reflect several factors, including local adiponectin production within oral tissues and limited transudation from plasma to saliva.

In addition, the dissociation observed between plasma and salivary adiponectin highlights the complexity of adipokine regulation in vivo. This evidence underscores the need for further validation studies that directly compare the kinetics of plasma and salivary adiponectin.

This study presents some limitations. First, the sample size was small. The final sample size (*n* = 9) did not reach the a priori target, which limits precision and increases the risk of type II error, particularly for between-protocol contrasts. To quantify the implications of this constraint, we performed sensitivity analyses (G*Power 3.1) to estimate minimum detectable effects. For the within-session omnibus time effect across four repeated measures (α = 0.05, two-tailed; power = 0.80; *n* = 9; assumed r = 0.50; ε = 1.0), the minimum detectable effect was f = 0.42, indicating that the design was primarily sensitive to medium-to-large time effects. For between-protocol comparisons, we considered the four paired contrasts ETS1 vs. ETS2 at matched time points; under a conservative family-wise α = 0.0125 (0.05/4) and power = 0.80, the minimum detectable paired effect was d_z = 1.39, indicating sensitivity primarily to large between-protocol differences. Accordingly, statistically non-significant ETS1 vs. ETS2 findings should be interpreted as inconclusive for small-to-moderate effects rather than evidence of equivalence, and the results should be considered preliminary [30].

Beyond sample size, there are additional limitations, such as the inclusion of male participants only because adiponectin regulation is known to be sex-dependent, and women generally exhibit higher basal adiponectin levels. The inclusion of only males was mainly driven by practical and methodological considerations related to the pilot nature of the study. Furthermore, other biomarkers such as inflammatory cytokines and metabolic signals (e.g., IL-6, IL-8, myostatin, glycolytic and lipid markers) were not detected [31]. In addition, the time point of adiponectin assessment considered in this study may not allow for evaluating the fluctuations occurring within the first hours post-exercise. Lastly, the lack of time-matched resting controls should also be considered in future studies.

On the other hand, this study presents several methodological and conceptual strengths. First, it addresses a well-defined and underexplored research gap by investigating the regulation of adiponectin in response to exercise protocols specifically differing in TUT, thus highlighting metabolic stress as a key modulator independent of external load. Second, integration of an adipose tissue marker with indices of muscle damage and perceived soreness provides a more comprehensive characterization of the stress–recovery response. Furthermore, the evaluation of adiponectin oligomers is noteworthy, since the high-molecular-weight oligoforms are considered the most biologically active. Finally, plasma and salivary adiponectin measurements highlight the compartment-specific regulation of serum adipokines after resistance exercise, but possibly the absence of salivary adiponectin.

However, further studies, including larger, gender-balanced cohorts, are needed to fully understand adiponectin as a mediator of exercise-induced metabolic adaptation. Furthermore, studies integrating inflammatory cytokines, myokines, and primary metabolic and inflammatory stressors will be useful to better define the relationship between adiponectin and muscle damage post-exercise recovery processes. Finally, collecting biological samples at an additional timing in the 6–24 h recovery interval will better characterize the timing of exercise-induced adiponectin modulation.

## 5. Conclusions

In conclusion, this study demonstrates that acute resistance exercise induces a time-dependent reduction in circulating adiponectin, regardless of tension time manipulation. The observed reduction in adiponectin, concomitant with increased creatine kinase and correlated with perceived muscle soreness, suggests that this decline reflects the presence of an adaptive metabolism in response to exercise acute stimulus. Future studies that consider long-term resistance training interventions and integrate other molecular markers should clarify the role of adiponectin within the muscle–adipose endocrine axis.

## Figures and Tables

**Table 1:** Participant’s anthropometric characteristics.

Variables	Mean	SD
Age (yrs)	23.9	3.0
Height (cm)	177.3	8.7
Weight (kg)	76.9	8.5
BMI (kg/m^2^)	24.5	2.3
Phase Angle (PhA) (°)	7.3	0.3
FFM (% of body weight)	85.2	2.7
FM (% of body weight)	14.8	2.7
Skeletal Muscle Mass (SMM, % of body weight)	44.5	3.5
Ratio BW/1RMLP	2.7	0.4
ACSM Relative Strength Percentile (1RM/BW)	88.9	3.3

Descriptive values are reported as mean and standard deviation. BMI, body mass index; FFM, fat-free mass; FM, fat mass; 1RM, one-repetition maximum; BW, body weight; ACSM Relative Strength Percentile (1RM/BW) indicates the normative percentile rank for the 1RM-to-body-mass ratio, based on the ACSM normative data for adult men.

**Table 2:** Exercise protocol.

Exercise	ETS1				ETS2			
	Set	Repetition	TUT	Rest	Set	Repetition	TUT	Rest
			(Sec. E-I-C-I)				(Sec. E-I-C-I)	
90° Leg Press	4	selected by participants, carrying out sets to muscular failure	5-1-2-1	90 s	4	selected by participants, ending each set with buffer of two repetitions in reserve (RIR)	2-1-2-1	90 s
Prone Last Pulldown								
Prone Leg Curl								
Seated Chest Press								
Leg Extension								
Seated Shoulder Press								
Standing Cable Triceps Extension	3				3			
Standing Biceps Curl (dumbbells)								

## Data Availability

The original contributions presented in this study are included in the article/Supplementary Material. Further inquiries can be directed to the corresponding author.

## Supplementary Materials

The following supporting information can be downloaded at: https://www.mdpi.com/article/10.3390/biom16020229/s1, Figure S1: Original Western Blot.
