# Supplementary material for: Acute Resistance Exercise Temporarily Reduces Circulating Adiponectin in Trained Young Men: A Pilot Study
Source: Biomolecules. 2026 Feb 2;16(2):229. doi: 10.3390/biom16020229 (PMC12938250; doi:10.3390/biom16020229)
Supplement: Supplementary file 1 [file biomolecules-16-00229-s001.zip › biomolecules-4057117-supplementary.pdf]

A

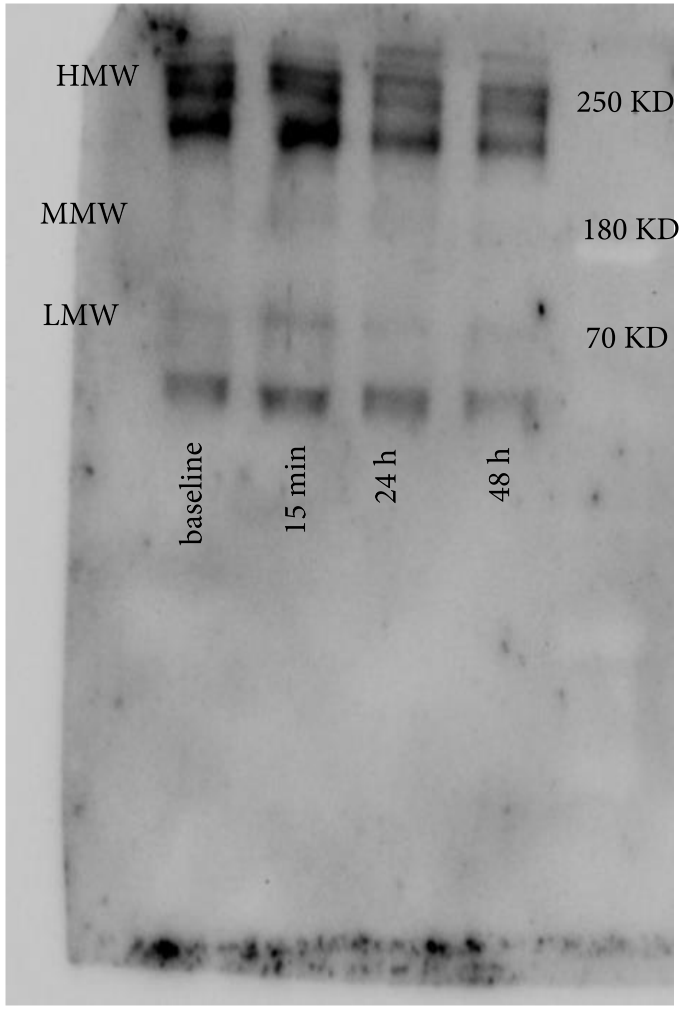

B

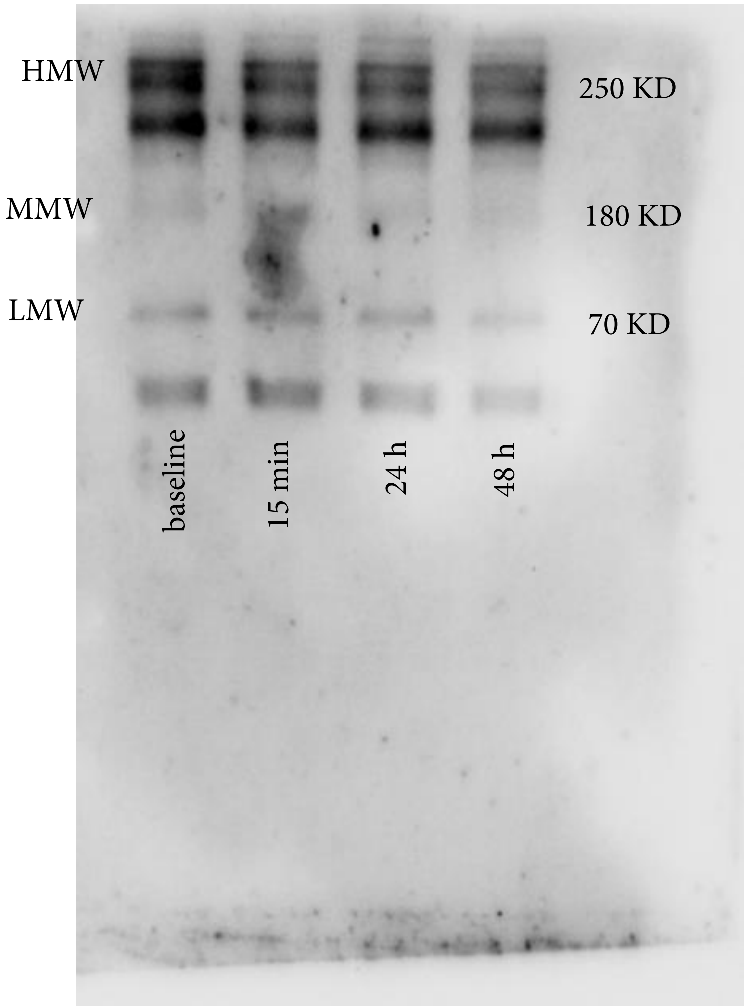

Figure S1. Original Western Blot. Western blotting analysis of adiponectin oligomers in plasma from subjects involved in the study at baseline and 15 min, 24 h, and 48 h after the training protocols. Representative Western blot images showing the oligomeric distribution of adiponectin [HMW (≥250 kDa), MMW (180 kDa), and LMW (70 kDa)] of one subject at baseline, 15 min, 24 and 48 hours post ETS 1 (A) and ETS 2 (B).
